# Supplementary material for: A Cryptic Site of Vulnerability on the Receptor Binding Domain of the SARS-CoV-2 Spike Glycoprotein
Source: bioRxiv. 2020 Mar 17:2020.03.15.992883. Preprint. [Version 1] doi: 10.1101/2020.03.15.992883 (PMC7217142; doi:10.1101/2020.03.15.992883)
Supplement: 1 [file NIHPP2020.03.15.992883-supplement-1.pdf]

bioRxiv preprint doi: https://doi.org/10.1101/2020.03.15.992883. The copyright holder for this preprint (which was not peer-reviewed) is the author/funder. This article is a US Government work. It is not subject to copyright under 17 USC 105 and is also made available for use under a CC0 license.

Table S1. Buried surface area of CR3022 in complex with SARS-CoV-2 RBD

CR3022 residue	Bond type	Accessible Surface Area (Å²)	Buried Surface Area (Å²)
H:VAL 2		19.25	0.17
H:TYR 27		196.06	37.22
H:GLY 28		34.09	22.53
H:ILE 30		79.49	45.95
H:THR 31		76.77	68.33
H:TYR 32		41.83	27.89
H:TRP 33		35.57	30.13
H:TYR 52	H	47.89	42.20
H:SER 55		51.50	17.77
H:GLU 56		75.85	18.52
H:LYS 73		111.52	9.17
H:GLY 95		8.55	8.55
H:SER 96		44.82	41.38
H:GLY 97		22.94	19.42
H:ILE 98		141.28	32.06
H:SER 99		105.91	25.84
H:THR 100		59.53	27.12
H:PRO 100A		109.00	11.16
H:ASP 101	H	89.73	32.30
H:VAL 102		34.00	0.17
L:TYR 27D	H	89.10	48.45
L:SER 27F	H	93.53	34.65
L:ILE 28		113.30	84.10
L:ALA 30		8.96	0.67
L:TYR 32		59.28	31.23
L:TYR 49		70.36	36.26
L:TRP 50		111.12	54.43
L:THR 53		57.72	22.88
L:ARG 54		77.45	9.69
L:GLU 55	H	92.85	32.34
L:SER 56		102.51	0.44

bioRxiv preprint doi: https://doi.org/10.1101/2020.03.15.992883. The copyright holder for this preprint (which was not peer-reviewed) is the author/funder. This article is a US Government work. It is not subject to copyright under 17 USC 105 and is also made available for use under a CC0 license.

**SARS-CoV-2 RBD residue**	**Bond type**	**Accessible Surface Area (Å²)**	**Buried Surface Area (Å²)**
A:TYR 369		89.63	43.79
A:ASN 370		109.19	26.31
A:PHE 374		39.48	7.06
A:SER 375		73.32	23.62
A:THR 376		34.77	7.37
A:PHE 377	H	70.41	64.16
A:LYS 378		81.20	66.58
A:CYS 379		47.55	44.69
A:TYR 380		56.89	44.21
A:GLY 381		77.51	75.98
A:VAL 382		27.99	25.04
A:SER 383		35.46	30.2
A:PRO 384		103.29	88.93
A:THR 385	H	109.91	63.9
A:LYS 386		112.50	65.4
A:LEU 390		47.38	22.93
A:PHE 392		19.18	5.95
A:ARG 408		159.50	5.51
A:PRO 412		34.93	1.0
A:ASP 428	H	114.22	33.26
A:PHE 429		16.72	2.10
A:THR 430		58.41	40.64
A:PHE 515		10.67	0.61
A:LEU 517	H	112.44	72.37
A:LEU 518		101.56	6.69

H: Hydrogen bond

bioRxiv preprint doi: https://doi.org/10.1101/2020.03.15.992883. The copyright holder for this preprint (which was not peer-reviewed) is the author/funder. This article is a US Government work. It is not subject to copyright under 17 USC 105 and is also made available for use under a CC0 license.

Table S2 | Protein DataBank files of SARS-CoV, MERS-CoV, and SARS-CoV-2 S structures

Virus	PDB Title	PDB ID	PMID	Release year	Type
SARS-CoV	Crystal structure of the SARS-CoV Spike protein fusion core	1WNC	15345712	2004	x-ray
	Structure of a proteolitically resistant core from the severe acute respiratory syndrome coronavirus S2 fusion protein	2BEZ	15604146	2004	x-ray
	Structure of a Proteolytically Resistant Core from the Severe Acute Respiratory Syndrome Coronavirus S2 Fusion Protein	2BEQ	15604146	2004	x-ray
	Post-fusion hairpin conformation of the sars coronavirus spike glycoprotein	1WYY	15840526	2005	x-ray
	Structure of SARS coronavirus spike receptor-binding domain complexed with its receptor	2AJF	16166518	2005	x-ray
	Crystal Structure of SARS-CoV Spike Receptor-Binding Domain Complexed with Neutralizing Antibody	2DD8	16597622	2006	x-ray
	Crystal Structure of anti-SARS m396 Antibody	2G75	16597622	2006	x-ray
	Crystal structure of SARS spike protein receptor binding domain	2GHV	16954221	2006	x-ray
	Crystal structure of SARS spike protein receptor binding domain in complex with a neutralizing antibody, 80R	2GHW	16954221	2006	x-ray
	Crystal structure of SARS spike protein receptor binding domain	2GHV	16954221	2006	x-ray
	X-ray crystal structure of the SARS coronavirus spike receptor binding domain in complex with F26G19 Fab	3BGF	19324051	2008	x-ray
	Crystal structure of spike protein receptor-binding domain from the 2005-2006 SARS coronavirus civet strain complexed with human-civet chimeric receptor ACE2	3D0I	18448527	2008	x-ray
	Crystal structure of spike protein receptor-binding domain from the 2002-2003 SARS coronavirus human strain complexed with human-civet chimeric receptor ACE2	3D0G	18448527	2008	x-ray
	Crystal structure of spike protein receptor-binding domain from the 2002-2003 SARS coronavirus civet strain complexed with human-civet chimeric receptor ACE2	3D0H	18448527	2008	x-ray
	Crystal structure of spike protein receptor-binding domain from SARS coronavirus epidemic strain complexed with human-civet chimeric receptor ACE2	3SCL		2012	x-ray
	Crystal structure of spike protein receptor-binding domain from a predicted SARS coronavirus civet strain complexed with human-civet chimeric receptor ACE2	3SCK		2012	x-ray
	Crystal structure of spike protein receptor-binding domain from a predicted SARS coronavirus civet strain complexed with human receptor ACE2	3SCJ		2012	x-ray
	Crystal structure of spike protein receptor-binding domain from a predicted SARS coronavirus human strain complexed with human receptor ACE2	3SCI		2012	x-ray
	SARS-CoV spike glycoprotein	5WRG	28008928	2017	EM
	Structure of SARS-CoV spike glycoprotein	5XLR	28008928	2017	EM
	Structure of the N-terminal domain (NTD)of SARS-CoV spike protein	5X4S	28393837	2017	x-ray
	Prefusion structure of SARS-CoV spike glycoprotein, conformation 1	5X58	28393837	2017	EM
	Prefusion structure of SARS-CoV spike glycoprotein, conformation 2	5X5B	28393837	2017	EM
	Tectonic conformational changes of a coronavirus spike glycoprotein promote membrane fusion	6B3O	29073020	2017	EM
	Crystal Structure of the Human Coronavirus SARS HR1 motif in complex with pan-CoVs inhibitor EK1	5ZVM	30989115	2019	x-ray
	SARS Spike Glycoprotein, Stabilized variant, C3 symmetry	6CRV	30356097	2018	EM
	SARS Spike Glycoprotein, Stabilized variant, single upwards S1 CTD conformation	6CRW	30356097	2018	EM
	SARS Spike Glycoprotein, Stabilized variant, two S1 CTDs in the upwards conformation	6CRX	30356097	2018	EM
	SARS Spike Glycoprotein, Trypsin-cleaved, Stabilized variant, C3 symmetry	6CRZ	30356097	2018	EM
	SARS Spike Glycoprotein, Trypsin-cleaved, Stabilized variant, one S1 CTD in an upwards conformation	6CS0	30356097	2018	EM

bioRxiv preprint doi: https://doi.org/10.1101/2020.03.15.992883. The copyright holder for this preprint (which was not peer-reviewed) is the author/funder. This article is a US Government work. It is not subject to copyright under 17 USC 105 and is also made available for use under a CC0 license.

	SARS Spike Glycoprotein, Trypsin-cleaved, Stabilized variant, two S1 CTDs in an upwards conformation	6CS1	30356097	2018	EM
	SARS Spike Glycoprotein - human ACE2 complex, Stabilized variant, all ACE2-bound particles	6CS2	30356097	2018	EM
	Trypsin-cleaved and low pH-treated SARS-CoV spike glycoprotein and ACE2 complex, ACE2-free conformation with three RBD in down conformation	6ACC	30102747	2018	EM
	Trypsin-cleaved and low pH-treated SARS-CoV spike glycoprotein and ACE2 complex, ACE2-free conformation with one RBD in up conformation	6ACD	30102747	2018	EM
	Trypsin-cleaved and low pH-treated SARS-CoV spike glycoprotein and ACE2 complex, ACE2-bound conformation 1	6ACG	30102747	2018	EM
	Trypsin-cleaved and low pH-treated SARS-CoV spike glycoprotein and ACE2 complex, ACE2-bound conformation 2	6ACJ	30102747	2018	EM
	Trypsin-cleaved and low pH-treated SARS-CoV spike glycoprotein and ACE2 complex, ACE2-bound conformation 3	6ACK	30102747	2018	EM
	Crystal Structure of the Human Coronavirus SARS HR1 motif in complex with pan-CoVs inhibitor EK1	5ZVM	30989115	2019	x-ray
	SARS-CoV complex with human neutralizing S230 antibody Fab fragment (state 1)	6NB6	30712865	2019	EM
	SARS-CoV complex with human neutralizing S230 antibody Fab fragment (state 2)	6NB7	30712865	2019	EM
	Crystal structure of anti- SARS-CoV human neutralizing S230 antibody Fab fragment	6NB8	30712865	2019	EM
MERS-CoV	Structure of the MERS-CoV fusion core	4MOD	24067982	2013	x-ray
	Crystal structure of MERS-CoV complexed with human DPP4	4L72	23835475	2013	x-ray
	Complex structure of MERS-CoV spike RBD bound to CD26	4KR0	23831647	2013	x-ray
	structure of the receptor binding domain (RBD) of MERS-CoV spike	4KQZ	23831647	2013	x-ray
	Bat-derived coronavirus HKU4 uses MERS-CoV receptor human CD26 for cell entry	4QZV	25211075	2014	x-ray
	Crystal structure of middle east respiratory syndrome coronavirus S2 protein fusion core	4NJL	24473083	2014	x-ray
	Receptor binding domain and Fab complex	4ZS6	26281793	2015	x-ray
	Complex structure of MERS-RBD bound with 4C2 antibody	5DO2	26391698	2015	x-ray
	Crystal structure of potent neutralizing antibody m336 in complex with MERS Co-V RBD	4XAK	26370782	2015	x-ray
	Structure of MERS-Coronavirus Spike Receptor-binding Domain (England1 Strain) in Complex with Vaccine-Elicited Murine Neutralizing Antibody D12 (Crystal Form 1)	4ZPT	26218507	2015	x-ray
	Structure of MERS-Coronavirus Spike Receptor-binding Domain (England1 Strain) in Complex with Vaccine-Elicited Murine Neutralizing Antibody D12 (Crystal Form 2)	4ZPV	26218507	2015	x-ray
	Structure of unbound MERS-CoV spike receptor-binding domain (England1 strain).	4ZPW	26218507	2015	x-ray
	Structure of the N-terminal domain (NTD) of MERS-CoV spike protein	5X4R	28393837	2017	x-ray
	Prefusion structure of MERS-CoV spike glycoprotein, three-fold symmetry	5X59	28393837	2017	EM
	Prefusion structure of MERS-CoV spike glycoprotein, conformation 1	5X5C	28393837	2017	EM
	Prefusion structure of MERS-CoV spike glycoprotein, conformation 2	5X5F	28393837	2017	EM
	Structure of MERS-CoV RBD in complex with a fully human antibody MCA1	5GMQ	28472421	2017	x-ray
	Crystal Structure of MERS-CoV S1 N-terminal Domain	5VYH	28807998	2017	x-ray
	Crystal Structure of MERS-CoV neutralizing antibody G4 Fab	5VZR	28807998	2017	x-ray
	MERS S ectodomain trimer in complex with variable domain of neutralizing antibody G4	5W9H	28807998	2017	EM
	MERS S ectodomain trimer in complex with variable domain of neutralizing antibody G4	5W9I	28807998	2017	EM
	MERS S ectodomain trimer in complex with variable domain of neutralizing antibody G4	5W9J	28807998	2017	EM

bioRxiv preprint doi: https://doi.org/10.1101/2020.03.15.992883. The copyright holder for this preprint (which was not peer-reviewed) is the author/funder. This article is a US Government work. It is not subject to copyright under 17 USC 105 and is also made available for use under a CC0 license.

	MERS S ectodomain trimer in complex with variable domain of neutralizing antibody G4	5W9K	28807998	2017	EM
	MERS S ectodomain trimer in complex with variable domain of neutralizing antibody G4	5W9L	28807998	2017	EM
	MERS S ectodomain trimer in complex with variable domain of neutralizing antibody G4	5W9M	28807998	2017	EM
	MERS S ectodomain trimer in complex with variable domain of neutralizing antibody G4	5W9N	28807998	2017	EM
	MERS S ectodomain trimer in complex with variable domain of neutralizing antibody G4	5W9O	28807998	2017	EM
	MERS S ectodomain trimer in complex with variable domain of neutralizing antibody G4	5W9P	28807998	2017	EM
	Crystal structure of Middle-East Respiratory Syndrome (MERS) coronavirus neutralizing antibody JC57-14 isolated from a vaccinated rhesus macaque in complex with MERS Receptor Binding Domain	6C6Y	29514901	2018	x-ray
	Crystal structure of Middle-East Respiratory Syndrome (MERS) coronavirus neutralizing antibody JC57-14 isolated from a vaccinated rhesus macaque.	6C6X	29514901	2018	x-ray
	Crystal structure of potent neutralizing antibody CDC2-C2 in complex with MERS-CoV S1 RBD	6C6Z	29514901	2018	x-ray
	Structural definition of a unique neutralization epitope on the receptor-binding domain of MERS-CoV spike glycoprotein	5YY5	29996104	2018	x-ray
	Structural definition of a unique neutralization epitope on the receptor-binding domain of MERS-CoV spike glycoprotein	5ZXV	29996104	2018	x-ray
	MERS-CoV complex with human neutralizing LCA60 antibody Fab fragment (state 1)	6NB3	30712865	2019	EM
	MERS-CoV S structure in complex with sialyl-lewisX	6Q05	31792450	2019	EM
	MERS-CoV spike N-terminal domain and 7D10 scFv complex	6J11	31296843	2019	x-ray
	MERS S0 trimer in complex with variable domain of antibody G2	6PZ8	31553909	2019	EM
	Crystal Structure of MERS-CoV neutralizing antibody G2 Fab	6PXG	31553909	2019	x-ray
	Crystal Structure of MERS-CoV S1-NTD bound with G2 Fab	6PXH	31553909	2019	x-ray
	MERS-CoV S structure in complex with 5-N-acetyl neuraminic acid	6Q04	31792450	2019	EM
	MERS-CoV S structure in complex with sialyl-lewisX	6Q05	31792450	2019	EM
	MERS-CoV S structure in complex with 2,3-sialyl-N-acetyl-lactosamine	6Q06	31792450	2019	EM
	MERS-CoV S structure in complex with 2,6-sialyl-N-acetyl-lactosamine	6Q07	31792450	2019	EM
	Complex structure of bat CD26 and MERS-RBD	6L8Q		2019	x-ray
	Crystal Structure of the Human Coronavirus MERS HR1 motif in complex with pan-CoVs inhibitor EK1	5ZVK	30989115	2019	x-ray
	Crystal structure of anti- MERS-CoV human neutralizing LCA60 antibody Fab fragment	6NB5	30712865	2019	EM
	MERS-CoV S complex with human neutralizing LCA60 antibody Fab fragment (state 2)	6NB4	30712865	2020	EM
SARS-CoV-2	Prefusion 2019-nCoV spike glycoprotein with a single receptor-binding domain up	6VSB	32075877	2020	EM
	Structure of the 2019-nCoV HR2 Domain	6LVN		2020	x-ray
	Structure of post fusion core of 2019-nCoV S2 subunit	6LXT		2020	x-ray
	Structural basis for receptor recognition by the novel coronavirus from Wuhan	6VW1		2020	x-ray
	The 2019-nCoV RBD/ACE2-B0AT1 complex	6M17		2020	x-ray
	Structure of the SARS-CoV-2 spike glycoprotein (closed state)	6VXX		2020	EM
	SARS-CoV-2 spike ectodomain structure (open state)	6VYB		2020	EM

bioRxiv preprint doi: https://doi.org/10.1101/2020.03.15.992883. The copyright holder for this preprint (which was not peer-reviewed) is the author/funder. This article is a US Government work. It is not subject to copyright under 17 USC 105 and is also made available for use under a CC0 license.


```
SARS-CoV-2-spike      SETKCTLKSFTVEKGIYQTSNFRVQPTESIVRFPNITNLCPFGEVFNATRFASVYAWNRK         356
batRs4231-spike       AELKCSVKSFEIDKGIYQTSNFRVAPSKEVVRFPNITNLCPFGEVFNATTFPSVYAWERK         343
batWIV16-spike        AELKCSVKSFEIDKGIYQTSNFRVAPSKEVVRFPNITNLCPFGEVFNATTFPSVYAWERK         343
SARS-CoV-spike        AELKCSVKSFEIDKGIYQTSNFRVVPSGDVVRFPNITNLCPFGEVFNATKFPSVYAWERK         343
                                                             ********** * *****:**

SARS-CoV-2-spike      RISNCVADYSVLYNSASFSTFKCYGVSPTKLNDLCFTNVYADSFVIRGDEVRQIAPGQTG         416
batRs4231-spike       RISNCVADYSVLYNSTSFSTFKCYGVSATKLNDLCFSNVYADSFVVKGDDVRQIAPGQTG         403
batWIV16-spike        RISNCVADYSVLYNSTSFSTFKCYGVSATKLNDLCFSNVYADSFVVKGDDVRQIAPGQTG         403
SARS-spike            KISNCVADYSVLYNSTFFSTFKCYGVSATKLNDLCFSNVYADSFVVKGDDVRQIAPGQTG         403
                      :**************: ********** ********:********::**:**********

SARS-CoV-2-spike      KIADYNYKLPDDFTGCVIAWNSNNLDSKVGGNYNYLYRLFRKSNLKPFERDISTEIYQAG         476
batRs4231-spike       VIADYNYKLPDDFLGCVLAWNTNSKDSSTSGNYNYLYRWVRRSKLNPYERDLSNDIYSPG         463
batWIV16-spike        VIADYNYKLPDDFTGCVLAWNTRNIDATQTGNYNYKYRSLRHGKLRPFERDISNVPFSPD         463
SARS-spike            VIADYNYKLPDDFMGCVLAWNTRNIDATSTGNYNYKYRYLRHGKLRPFERDISNVPFSPD         463
                       ************ ***:***:.. *:.  ***** ** .*:.:*.*:***:*.  :. .

SARS-CoV-2-spike      STPCNGVEGFNCYFPLQSYGFQPTNGVGYQPYRVVVLSFELLHAPATVCGPKKSTNLVKN         536
batRs4231-spike       GQSCSA-IGPNCYNPLRPYGFFTTAGVGHQPYRVVVLSFELLNAPATVCGPKLSTDLIKN         522
batWIV16-spike        GKPCTP-PAFNCYWPLNDYGFYITNGIGYQPYRVVVLSFELLNAPATVCGPKLSTDLIKN         522
SARS-spike            GKPCTP-PALNCYWPLNDYGFYTTTGIGYQPYRVVVLSFELLNAPATVCGPKLSTDLIKN         522
```

Fig. S1. Sequence alignment of the receptor binding domain of SARS-CoV and related betacoronaviruses measured by Biolayer Interferometry for reactivity to SARS-CoV antibodies.

bioRxiv preprint doi: https://doi.org/10.1101/2020.03.15.992883. The copyright holder for this preprint (which was not peer-reviewed) is the author/funder. This article is a US Government work. It is not subject to copyright under 17 USC 105 and is also made available for use under a CC0 license.

Fig. S2

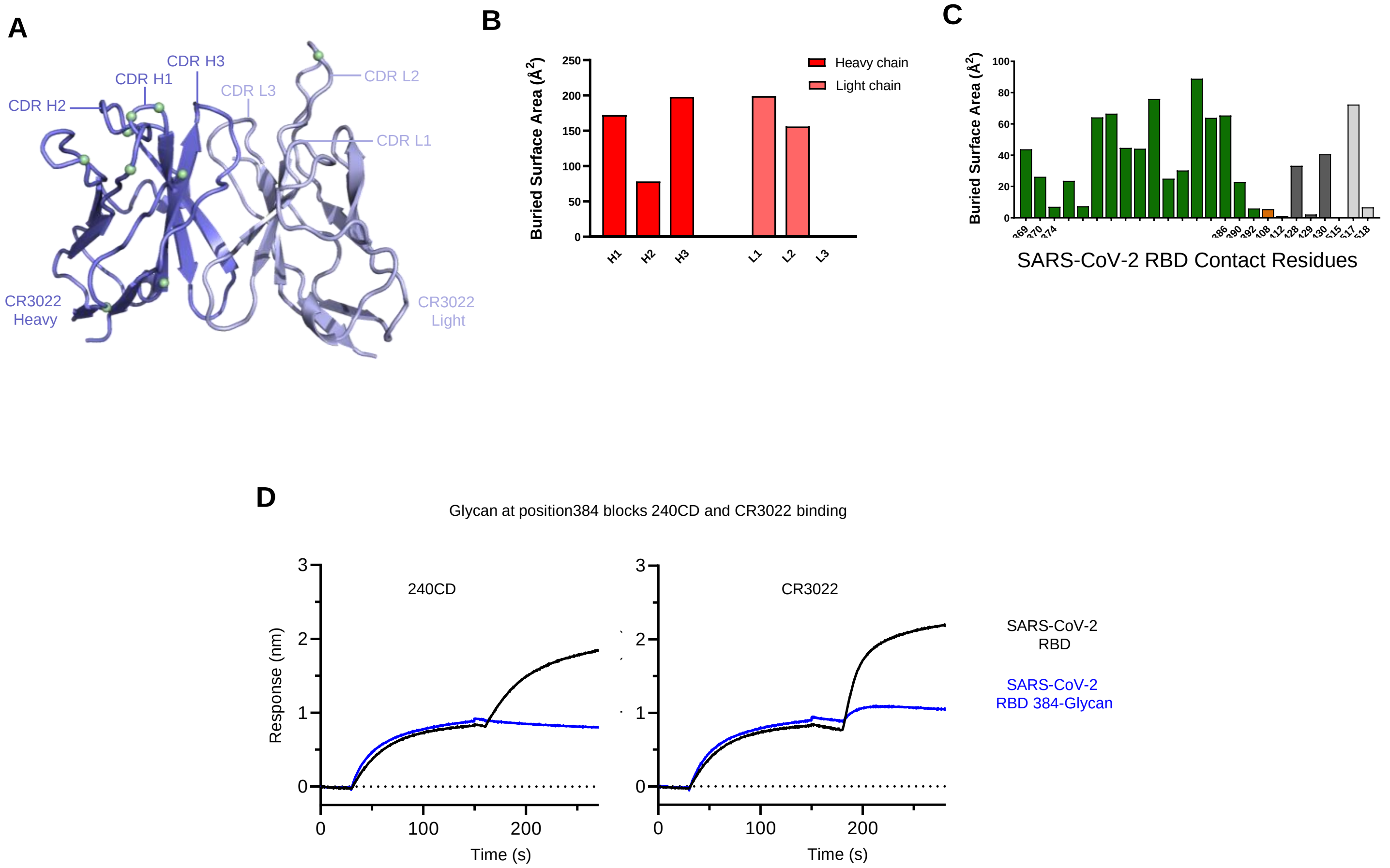

Fig. S2. CR3022 Fab crystal structure and epitope analysis. A CR3022 is shown in ribbon representation, with CDR loops indicated. Residues which have undergone somatic hypermutation are indicated with green spheres. **B** Heavy and light chain CDR loops paratope buried surface area. **C** Buried surface area analysis of the CR3022 epitope on the SARS-CoV-2 RBD. **D** Antibody binding to SARS-CoV-2 RBD and RBD with a glycan at position 384. RBD is initially loaded onto the HIS probe, followed by incubation with RBD variants.

bioRxiv preprint doi: https://doi.org/10.1101/2020.03.15.992883. The copyright holder for this preprint (which was not peer-reviewed) is the author/funder. This article is a US Government work. It is not subject to copyright under 17 USC 105 and is also made available for use under a CC0 license.

Fig. S3

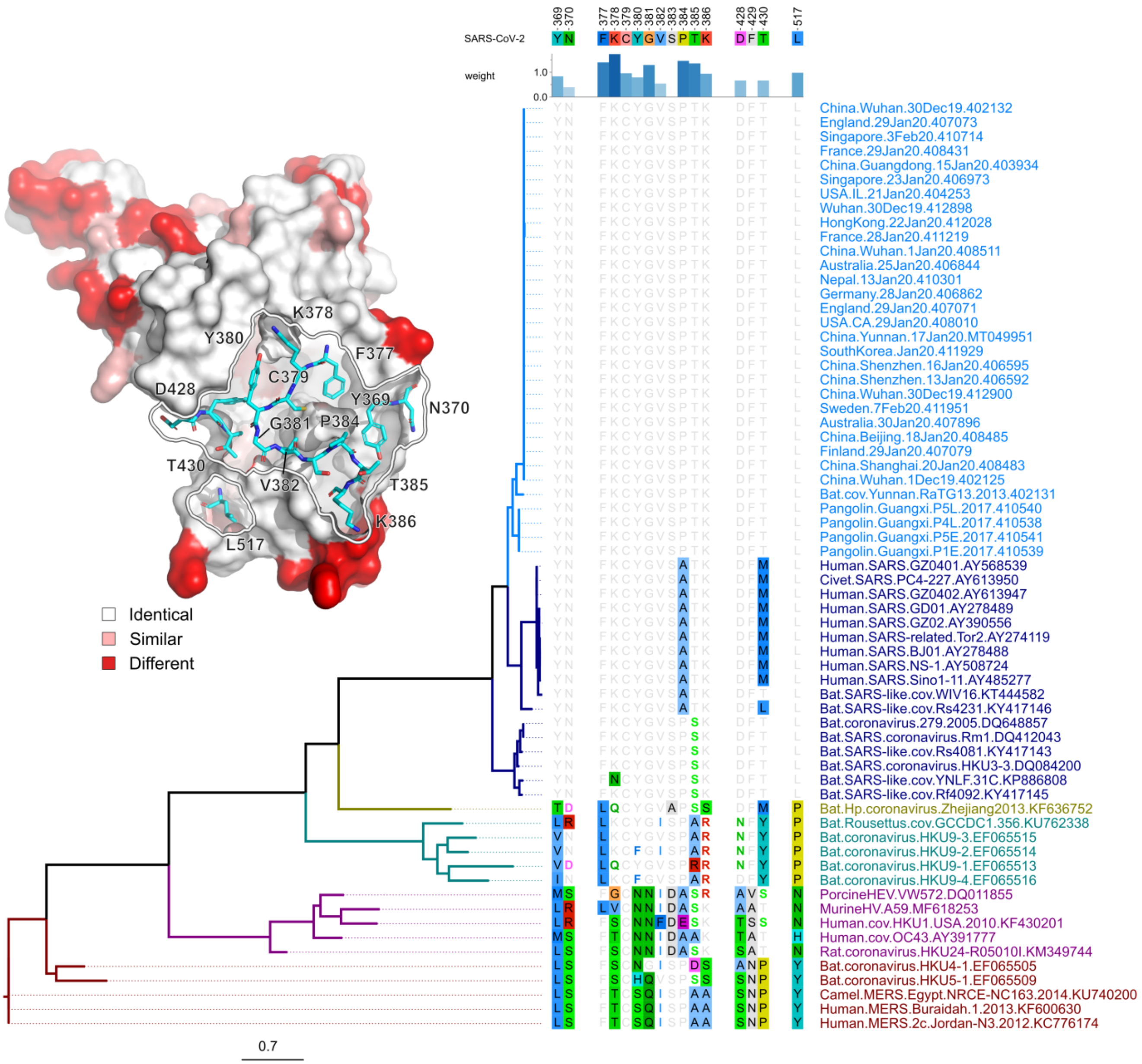

Fig. S3. Structural and sequence analysis of the CR3022 epitope. Analysis of the CR3022 footprint across betacoronaviruses. The CR3022 epitope on SARS-CoV-2 (China.Wuhan.30Dec19.402132) RBD is compared across betacoronaviruses. The epitope is numbered according to the Wuhan reference; the strength of the interaction between the Ab and the spike protein is indicated by the height and color of the histogram bars above the sequence alignment. Sequences are ordered based on their phylogenetic relationships based on a maximum likelihood phylogenetic tree derived from amino acid RBD sequences. The RBD structure is shown in surface representation and depicts mutations between SARS-CoV-1 and SARS-CoV-2 in red; the CR3022 epitope is outlined in white, with contact residues shown in stick representation.

bioRxiv preprint doi: https://doi.org/10.1101/2020.03.15.992883. The copyright holder for this preprint (which was not peer-reviewed) is the author/funder. This article is a US Government work. It is not subject to copyright under 17 USC 105 and is also made available for use under a CC0 license.

Fig. S4

A

```
                    340                 360                 380                 400                 420
                     |                   |                   |                   |                   |
CR3022    ITNLCPFGEVFNATRFASVYAWNRKRISNCVADYSVLYNSASFSTFKCYGVSPTKLNDLCFTNVYADSFVIRGDEVRQIAPGQTGKIADYNYKLPDDFTG
ACE2      ITNLCPFGEVFNATKFPSVYAWERKKISNCVADYSVLYNSTFFSTFKCYGVSATKLNDLCFSNVYADSFVVKGDDVRQIAPGQTGVIADYNYKLPDDFMG
80R       ITNLCPFGEVFNATKFPSVYAWERKKISNCVADYSVLYNSTFFSTFKCYGVSATKLNDLCFSNVYADSFVVKGDDVRQIAPGQTGVIADYNYKLPDDFMG
230S      ITNLCPFGEVFNATKFPSVYAWERKKISNCVADYSVLYNSTFFSTFKCYGVSATKLNDLCFSNVYADSFVVKGDDVRQIAPGQTGVIADYNYKLPDDFMG
F26G19    ITNLCPFGEVFNATKFPSVYAWERKKISNCVADYSVLYNSTFFSTFKCYGVSATKLNDLCFSNVYADSFVVKGDDVRQIAPGQTGVIADYNYKLPDDFMG
M396      ITNLCPFGEVFNATKFPSVYAWERKKISNCVADYSVLYNSTFFSTFKCYGVSATKLNDLCFSNVYADSFVVKGDDVRQIAPGQTGVIADYNYKLPDDFMG
          **************:*.*****.**:**************: ********** ********:********::**:********** ************ *
                    440                 460                 480                 500                 520
                     |                   |                   |                   |                   |
CR3022    CVIAWNSNNLDSKVGGNYNYLYRLFRKSNLKPFERDISTEIYQAGSTPCNGVEGFNCYFPLQSYGFQPTNGVGYQPYRVVVLSFELLHAPATVCGPKKST
ACE2      CVLAWNTRNIDATSTGNYNYKYRYLRHGKLRPFERDISNVPFSPDGKPCTP-PALNCYWPLNDYGFYTTTGIGYQPYRVVVLSFELLNAPATVCGPKLST
80R       CVLAWNTRNIDATSTGNYNYKYRYLRHGKLRPFERDISNVPFSPDGKPCTP-PALNCYWPLNDYGFYTTTGIGYQPYRVVVLSFELLNAPATVCGPKLST
230S      CVLAWNTRNIDATSTGNYNYKYRYLRHGKLRPFERDISNVPFSPDGKPCTP-PALNCYWPLNDYGFYTTTGIGYQPYRVVVLSFELLNAPATVCGPKLST
F26G19    CVLAWNTRNIDATSTGNYNYKYRYLRHGKLRPFERDISNVPFSPDGKPCTP-PALNCYWPLNDYGFYTTTGIGYQPYRVVVLSFELLNAPATVCGPKLST
M396      CVLAWNTRNIDATSTGNYNYKYRYLRHGKLRPFERDISNVPFSPDGKPCTP-PALNCYWPLNDYGFYTTTGIGYQPYRVVVLSFELLNAPATVCGPKLST
          **:***:.*:*:.  ***** ** :*:.:*:*******.  :. ...**.   .:***:**:.***  *.*:*************** ********* **
```

B

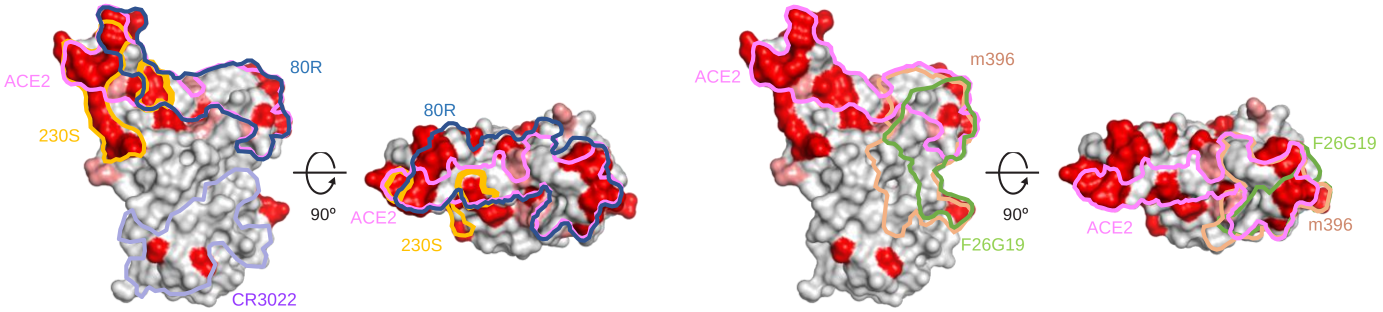

C

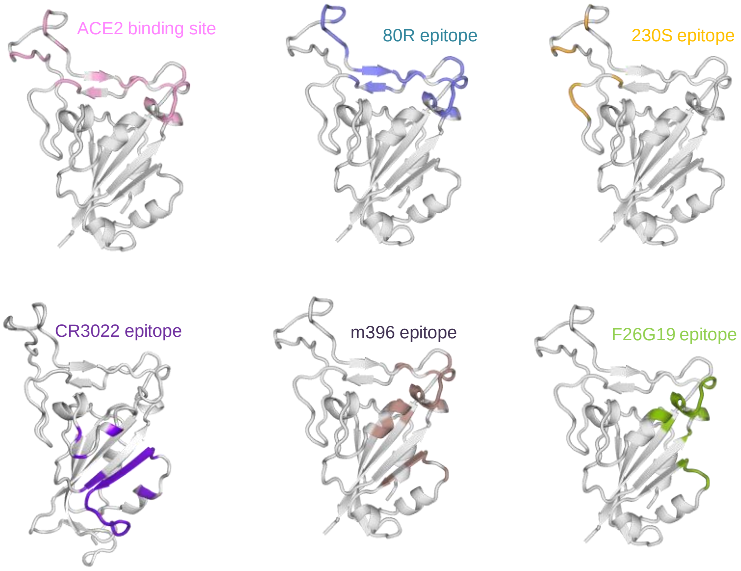

Fig. S4. SARS-CoV-2 and SARS-CoV antibody epitope analysis. A Sequence alignment of SARS-CoV-2 and SARS-CoV RBD. Antibody and ACE2 receptor interacting residues are highlighted on the sequence. **B** Antibody epitopes and receptor binding sites are outlined on the surface of the SARS-CoV RBD structures. The Sequence differences between SARS-COV-2 and SARS-CoV are indicated by red coloring on the RBD surface. **C** Antibody epitopes are shown on the SARS-CoV RBD molecule depicted in ribbon representation. The CR3022 epitope is shown on the SARS-CoV-2 RBD molecule.
